# Inflammation of the Uterus

**Published:** 1867-09

**Authors:** Joseph Kammerer, B. F. Dawson

**Affiliations:** New York; New York


### SELECTIONS.

*Inflammation of the Uterus.* (*Being a Translation from the German of "Klob on the Pathological Anatomy of the Female Sexual Organs.*") By JOSEPH KAMMERER, M. D., and B. F. DAWSON, M. D., New York.

The inflammatory processes to which the uterus is subject, affect either its *muscular substance, its mucous lining, or its peritoneal covering.* The latter will be discussed with anoma lies of the uterine ligaments and peritoneum.

*Inflammation of the Muscular Substance of the Uterus, Metritis.*

Inflammation of the substance of the non-gravid uterus seems to be one of the rarest affections to which this organ is liable and if some uterine pathologists doubt the existence of such a disease, and explain the cases diagnosed as metritis, as cases of perimetritis, pathological anatomy, considering the small number of semi-authenticated post mortem cases, must pronounce upon it with some reservation. I have not met with a single case, which with any degree of certainty, I could pronounce to be one of jenuine metritis, and I therefore borrow the following description from other authors:

*In acute parenchymatous metritis* the uterus, especially in its upper third, is found to be enlarged (even to the size of a goose's egg,) thickened anteriorly and posteriorly, and reddish or bluish red, in some places more than others. The substance of the uterine walls is very succulent, and marked with small extravasations, and a viscid fluid can be expressed from it containing free nuclei and a small quantity of pus corpuscles. In many the uterine tissue may be so softened as to occasion larger extravasations with destruction of tissue. The mucous membrane of the fundus and body is vascular, reddened and relaxed; that of the cervix is generally normal. The vaginal portion of the uterus is tumefied, œdematous and eroded, and the papillæ are sometimes distinctly prominent.

The most obvious alterations in the inner layers of the uterine substance resulting from acute parenchymatous metritis occur in that portion of the organ which contains the largest amount of *connecting tissue;* the inflammatory action generally extends outwards, giving rise to *perimetritis* and *pelvic peritonitis*, and is frequently combined with *encolpitis*, *metrosalpingitis*, and *oophoritis.*

*Acute parenchymatous metritis* may terminate, 1st, in resolution with absorption of the exudation and a return of the uterus to its normal size; 2d, in consequence of the inflammatory action, proliferation of connective tissue may ensue resulting in permanent enlargement or induration of the substance of the uterus; 3d, as it is incorrectly stated, acute metritis may become chronic, and chronic engorgement be developed.

Kiwisch makes three distinct forms of parenchymatous metritis; 1st, metritis with œdema of the uterus, which according to his description may be considered as hyperæmia with intumescense from transudation; 2d, metritis with increased firmness of tissue, or acute infarctus of the uterus, and finally, 3d, hæmorrhagic metritis.*

A futher termination of parenchymatous metritis is the extremely rare formation of an abscess in the substance of the uterus. Bartholin's observation (the uterus of a girl 13 years old, filled with ulcers) does not seem to belong to this class; but Reinmann (in Voigtel's work) describes an abscess of the uterus which opened externally through the abdominal walls. Scanzoni also observed an abscess, the size of a goose's egg, in the right circumference of the fundus uteri, which ruptured into the peritoneal cavity. Bird (*Lancet*, Eeb. 1844,) describes a case in which an abscess situated in the posterior wall of the uterus, opened into the rectum.

The directions in which a uterine abscess may perforate, vary, of course, according to its situation; it may open inwardly into the uterine or vaginal cavity, or outwardly. If adhesions exist

---

* Any one unprejudiced must be struck with the uncertainty of the great gynæcologist in his description of metritis and his rather unsuccessful attempt at classifying it. His description of serous metritis is deficient of all anatomical requisites of inflammation; in "acute infarctus," an analogy to chronic infarctus was intended, which latter he was unwilling to drop; for he (Kiwisch) says, that the more acute the affection (that is, metritis with increased firmess of tissue) the more relaxed the uterine tissue is found. Finally, hæmorrhagic metritis is nothing else but acute uterine catarrh with hæmorrhage.

between the uterus and neighboring organs, the abscess may perforate externally through the anterior abdominal wall, or into the bladder, cæcum, ileum, and sigmoid flexure of the colon, or the pus may burrow between the folds of the broad ligaments into encysted portions of the abdominal cavity (recto-uterine or vesico-uterine spaces;) or lastly, it may pass directly into the peritoneal cavity, which latter occurrence is always followed by general peritonitis. A uterine abscess may also cause death from metastatic processes, or the long duration of the purulent secretion may exhaust the patient.

Acute parenchymatous metritis generally arises from acute catarrh of the uterus.

### *Inflammation of the Mucous Membrane of the Uterus.*

The inflammatory processes occurring in the non-gravid uterus are various, and we may distinguish as the chief forms of such processes, *catarrhal* and *croupy* inflammation. To this I will add the anatomical description of the so-called *membranous dysmenorrhœa*, as I am persuaded that this morbid process is more an inflammatory derangment than any other. Catarrhal inflammation of the uterus, as elsewhere, is divided into the acute and chronic form.

### *Acute Catarrhal Inflammation of the Uterus.*

Acute catarrh (catarrhal endometritis) affect the whole mucous membrane of the uterus, but chiefly that of its body and fundus, whilst that of the cervical canal is rarely affected.

In this affection the mucous membrane of the body and fundus uteri may be so intensely injected as to appear dark red, tumefied and velvet like; the utricular glands, however, are not so much elongated as during menstrual fluxion; the membrane is also so softened that it may readily be removed, or scraped off with the handle of a scalpel. In the higher degrees of this disease small round striated extravasations are seen scattered over the mucous membrane as dark red spots.

The mucous membrane of the cervix uteri is more injected than swollen where it covers the turgid follicles; that of the vaginal portion of the uterus is generally of a darker red. In virgins the os uteri is transformed into a small round depression, owing to the tumefaction of the vaginal portion, the mucous follicles of its lips are enlarged and frequently have small erosions between them, and the papillæ of the vaginal portion are visible to the naked eye, especially at the edges of the above mentioned erosions.

The whole uterus generally appears to be increased in substance, and its tissue more vascular and succulent, especially in the layers nearest the mucous membrane. The cervix, beyond increased succulence, hardly exhibits any alteration, whilst the vaginal portion is hyperæmic, tumefied œdematous, and sometimes of a spongy softness.

At the outset of inflammation, the mucous membrane of the body and fundus uteri secretes a thin clear mucus, which, as the inflammation progresses, becomes viscid, thick and turbid from the admixture of desquamated epithelium. In regard to the latter, it is necessary to state that in many cases the glandular utricular follicles cast off their entire cellular coverings, which latter are found in the mucus as collapsed casts. Nylander and Virchow have observed a similar expulsion of the whole contents of glands during menstruation, and I have repeatedly found the same in various tumefications of the uterine mucous membrane. Finally, the color of the secretion changes to yellowish or yellow, and from the admixture of purulent elements it becomes cream-like.

Uterine pathologists assert that chronic uterine catarrh is generally associated with derangments of menstruation, and that conception is not impossible, but rarely occurs when it exists. It is an interesting observation that females who have suffered for a long time from blennorrhœa have a pre-disposition to the occurence of *placenta prœvia.*

The frequency of chronic catarrhal endemetritis being complicated with chlorosis, scrofula, tuberculosis and diseases of the heart, is a fact universally admitted, and the profuse secretion and purulent discharge contributes not a little to the complete exhaustion of the patient. In scrofulous and tuberculosus girls, chronic uterine catarrh generally sets in at the period of puberty, and is combined with amennorrhœa. As a sequel to the catarrh in such cases, the various proliferations of the mucous membrane rarely occur; but the catarrh sometimes preceeds tuberculosis of the uterus.

It is different with the secretion of the cervical portion; its glands at the outset of the inflammation undoubtedly secrete a larger quantity of, and a thicker mucus. Nabothian vesicles are developed and the fluid contained in them presents the turbid cloudy appearance previously mentioned, finally becoming whitish or white. If the inflammatory process increases in intensity, the mucus becomes deliquescent, and on opening such a vesicle its entire contents flow out like water in which the cloudy turbescence appears in streaks. These vesicles, however, burst spontaneously, and the hypersecretion of the cervix becomes very fluid and finally purulent. In no other secretion do we so frequently and distinctly observe a so-called *cellular halo* (cells having no investing membrane.) Kölliker and Scanzoni sometimes also found a few fungi with round branches, similar to those seen in fermenting liquids, and isolated vibriones. I must not omit mentioning that the cell of the secretion are often disposed in rows like strings of beads.

Acute catarrhal endemotritis rarely or never occurs before puberty; after that time, however, it is quite frequent. Duparcque states that, in females who have sexual intercouse, the mucous membrane of the cervical canal is always the first portion affected, and that from thence it spreads to the mucous membrane of the body of the uterus.

As causes of this affection we find mentioned, colds taken during menstruation, excesses in drink and sexual intercourse, infection with gonorrhœal virus (virulent catarrh,) and other diseases such as typhus, dysintery, cholera, general tuberculosis, and diseases of the heart (metastatic constitutional catarrh, *Kiwisch.*)

Acute catarrh has a tendency to extend to the oviducts, and undoubtedly from them to the peritoneum; it sometimes also causes inflammation of the peritoneum independent of any such process in the oviducts. It may extend downwards to the vagina, unless it has originated there and extends upwards to the uterus; acute parenchymatous metritis, as previously mentioned, may also arise from it.

This affection may terminate in resolution, but in the majority of cases it passes into the chronic form.

The so-called hydrorrhœa of pregnant females is considered by some to be catarrh of the gravid uterus, and it seems reasonable to suppose that a portion of the uterine mucous membrane, unlike the rest, may not be transformed into a decidua and consequently cause increased transudation from the hyperæmia connected with pregnancy.

*Chronic Catarrh of the Uterus.*

Chronic catarrh of the uterus, a condition frequently met with, is characterized by a constant irritation of the mucous membrane of the uterus often combined with considerable hypersecretion.

The mucous membrane of the body and fundus uteri is generally swollen, but not always highly congested in the dead body; on the contrary, rather pale, and especially when it is considerably intumesced, of a bluish gray color. We find scattered throughout it numerous dots or specks of pigment, generally gray or blackish gray, but rarely of a rusty dark brown color. The surface of the membrane is either smooth, or papillary and uneven, the latter being especially the case at the posterior wall, which is sometimes covered with various secretions, and growths resembling granulations. The mucous membrane is also generally softened and more succulent, but can rarely be separated from the uterine wall in as large pieces as in acute catarrh.

The cervical mucous membrane is likewise injected at various points and covered with viscid secretions; Nabothian glands are numerously developed and exceedingly distended. The transverse folds are intumesced and sometimes even œdematous. The vaginal portion is frequently enlarged, its tissue in a state of spongy relaxation, and its external surface affected with papillary hypertrophy. On its inner surface the swollen mucous follicles are prominent, and the os frequently dilated. In the majority of cases the latter is surrounded with excoriations and even with granulating ulcers.

The secretiuo in some cases of chronic uterine catarrh is often enormous (blennorrhœa,) but in others it is slight, there is generally, however, a marked hypersecretion. The mucus secreted is turbid or even purulent in various degrees, but rarely mixed with blood (excepting shortly before or after menstruation) (*Scanzoni.*)

The uterine substance is either affected with a diffuse growth of connective tissue, in consequence of which it becomes denser and firmer, or, it is flaccid and markedly atrophied. In the latter case the cavity of the organ is often much distended, especially in those cases where the cervical canal is occluded by the well known glassy mucus.

When chronic catarrh is of long duration, the mucous membrane, especially that of the body and fundus, undergoes important anatomical changes; its glands, either from constriction or atrophy of their superior portions, frequently change into small cysts, or are cast off, which latter occurrence, especially when the cavity of the uterus is distended, gives the mucous membrane a net-like appearance.

The ciliary epithelium which was cast off at the outset of the disease has been replaced by cylindrical epithelium; finally this also is cast off and *polymorphous lining cells*, which can hardly be called true basement epithelium, occupy its place. In some cases we also notice a desquamation of the epithelium, erosions, and small, smooth lined depressions evidently caused by the rupture of small cysts. It is probably owing to this development and rupture of cysts that the delicate ridge-like elevations are formed, especially at the internal orifice, which give rise to adhesions.

While, as above described, the epithelium is transformed and the glands become atrophied, the mucous membrane also becomes thin, and finally is replaced by a thin layer of connective tissue, which is covered by the polymorphous cells mentioned. More rarely we find the mucous membrane transformed into a callous stratum varying in thickness and attached to the submucous connective tissue, and there in the above mentioned stratum we find small cysts which are the remains of degenerated glands (*Rokitansky.*)

More frequently the dense sub-mucous stratum, especially at the borders of the internal orifice, becomes atrophied and Nabothian vesicles are developed in it, thus causing a predisposition to uterine flexion.

The consequences of chronic catarrh of the uterus have already been described; they are, circumscribed proliferations of the mucous membrane, glandular and cystic polypi, also fibrous polypi when the sub-mucous tissue has a tendency to proliferate; perhaps also fibroid tumors will be developed if the formative action is sufficiently increased. After the development of such growths their presence seems to occasion a constant irritation and thereby favors the continuance of the chronic catarrh. Hydrometra and hæmatometra may also be developed in consequence of adhesions.

Chronic uterine catarrh generally proceeds from acute catarrh. and sometimes occurs in consequence of the puerperal state. It is also readily developed in cachectic women, and lastly, may be caused by the virus of gonorrhœ. In young women and prostitutes it is said to occur as a consequence of masturbation. It is said to extend down to the vagina and up to the oviducts, and in the latter case especially, it leads so serious consequences; sometimes, however, it originates in the vagina and spreads by continuity.

